# ADHD medications use and risk of mortality and unintentional injuries: a population-based cohort study

**DOI:** 10.1038/s41398-024-02825-y

**Published:** 2024-02-28

**Authors:** Helen-Maria Vasiliadis, Carlotta Lunghi, Elham Rahme, Louis Rochette, Martin Gignac, Victoria Massamba, Fatoumata Binta Diallo, Alvine Fansi, Samuele Cortese, Alain Lesage

**Affiliations:** 1 Department of Community Health Science, Faculty of Medicine and Health Sciences, University of Sherbrooke, 150 Place Charles-Le Moyne, Longueil, QC J4K 0A8 Canada; 2 Centre de Recherche Charles-Le Moyne, 150 Place Charles-Le Moyne, Longueil, QC J4K 0A8 Canada; 3 Department of Health Sciences, Université du Québec à Rimouski, 1595 Boulevard Alphonse-Desjardins, Lévis, QC G6V 0A5 Canada; 4 Department of Medical and Surgical Sciences, University of Bologna, Via Irnerio, 48 - 40126 Bologna, Italy; 5 Institut National de Santé Publique du Québec (National Public Health Institute of Quebec), 945, avenue Wolfe, Quebec, QC G1V 5B3 Canada; 6 Department of Medicine, Division of Clinical Epidemiology, McGill University, 5252 de Maisonneuve Blvd, Montreal, QC H4A 3S5 Canada; 7 Montreal Children’s Hospital, McGill University Montreal, 1001 Décarie Blvd, Montréal, QC H4A 3J1 Canada; 8 Centre intégré universitaire de santé et de services sociaux de l’Ouest-de-l’Île-de-Montréal /Montreal West Island Integrated University Health and Social Services Centre, Montreal, QC Canada; 9 Centre for Innovation in Mental Health, School of Psychology, Faculty of Environmental and Life Sciences, University of Southampton, Highfield Campus, Building 44, Room 4059, University Rd, Southampton, SO171PS UK; 10 Clinical and Experimental Sciences (CNS and Psychiatry), Faculty of Medicine, University of Southampton, Southampton, UK; 11 Solent NHS Trust, HighPoint Venue, Bursledon Rd, Southampton, SO19 8BR UK; 12 Hassenfeld Children’s Hospital at NYU Langone, New York University Child Study Center, One Park, New York City, NY 10016 USA; 13 Department of Precision and Regenerative Medicine and Ionian Area (DIMEPRE-J), University of Studies of Bari “Aldo Moro”, Bari, Italy; 14 Department of Psychiatry and Addictology, University of Montreal, Research Centre of the Institut universitaire en santé mentale de Montréal, 7401, rue Hochelaga, Montreal, QC H1N 3M5 Canada

**Keywords:** ADHD, Scientific community

## Abstract

We assessed the association between the use of medications for attention-deficit/hyperactivity disorder (ADHD) and the risk of all-cause mortality and unintentional injuries leading to emergency department (ED) or hospital admission in individuals aged ≤24 years with ADHD. We conducted a population-based retrospective cohort study between 2000 and 2021 using Quebec health administrative data. Individuals were followed from the first ADHD diagnosis or ADHD medication claim until turning 25, death, or study end. Exposure was defined as mutually exclusive episodes of ADHD medication use and/or coverage under the public provincial drug plan (PDP): 1) covered and not treated with ADHD medication; 2) covered and treated with ADHD medication; and 3) not covered under the PDP. The risk of all-cause mortality and unintentional injuries associated with exposure episodes was estimated using multivariable survival analyses. The cohort included *n* = 217 192 individuals aged 1–24 years with a male to female ratio of close to 2:1. Compared to non-medication use, episodes of ADHD medication use, overall, were associated with reduced all-cause mortality (adjusted hazard ratio, aHR 0.61, 95% CI 0.48–0.76) and unintentional injury leading to ED (0.75, 0.74–0.77) or hospitalisation (0.71, 0.68–0.75). Episodes of stimulants were associated with a lower risk of all-cause mortality and reduced risk of unintentional injuries, while episodes with non-stimulants and with both stimulants and non-stimulants concomitantly were associated with reduced risk of unintentional injuries, but not of all-cause mortality. Although residual confounding cannot be excluded, stimulants may have a protective effect in terms of risk of all-cause mortality and both stimulants and non-stimulants for ADHD may reduce the risk of unintentional injuries. The findings of the current study should inform clinical decision making on the choice of starting a pharmacological treatment for ADHD, when a balance needs to be struck between expected benefits and possible risks.

## Introduction

Attention-deficit/hyperactivity disorder (ADHD) is the most common neurodevelopmental condition in childhood and adolescence, with an estimated worldwide prevalence of around 5−7% [1]. Its impairing symptoms persist in adulthood in about 2.5% of childhood cases [2]. ADHD has been associated with psychiatric comorbidity and physical conditions, including metabolic, nervous system, respiratory, and musculoskeletal diseases, obesity [3–6], substance use disorders [7] as well as with impaired sleep [8]. There is also evidence of an increased risk of premature mortality in individuals with ADHD [9], accounted for, in part, by an increased risk of unnatural causes, including unintentional injuries and accidents [10–12]. Although the severity of ADHD symptoms and the rates of psychiatric comorbidity are similar among boys and girls [13], the sex differences in prevalence of ADHD diagnosis and treatment are in part explained by increased clinical referral rate and detection of ADHD in boys as compared to girls [14]. In a Swedish population cohort study of twins, significant sex-symptom interactions were also observed, where the effect of the presence of hyperactive/impulsive and conduct symptoms on receipt of a diagnosis of ADHD and treatment was stronger in girls than in boys [15]. Children and adolescents with ADHD had twice the medical consultations and hospitalizations than matched controls leading up to their diagnosis, suggesting the opportunity for earlier detection and treatment [16]. Socioeconomic factors have also been associated with ADHD diagnosis and treatment, with children in single-parent homes and without health insurance less likely to be assessed and receive treatment [17]. Regional variations in ADHD diagnosis and treatment have also been reported in public health systems [18–21].

In short-term randomised controlled trials, ADHD medications - stimulants and non-stimulants-have been found efficacious in reducing ADHD core symptoms, with larger effect sizes - at the group level - for stimulants, and overall well tolerated [22]. Observational studies have shown additional benefits of ADHD medications in real-world settings on important outcomes from a public health standpoint, such as the reduction of criminal acts, car accidents, and substance abuse [23]. Furthermore, two studies have explored the association between ADHD medication use and the risk of all-cause mortality. However, one focused on methylphenidate only and did not consider time-varying exposure to combinations of ADHD medications [24], while the other one did not include a control group nor a self-controlled methodology (i.e., assessing the outcome in the same individual with and without medication) [25].

Evidence from studies using a self-controlled methodology has also shown a protective effect of ADHD medications on the risk of unintentional traumatic injuries [26–29]. However, it is currently unknown whether stimulants and non-stimulants affect the risk of unintentional injuries differently. As such, it is important to assess the effects of these two classes of medications separately, given their different efficacy on ADHD core symptoms and mechanism of action [30].

Therefore, the association between stimulant and non-stimulant use and the risk of all-cause mortality and unintentional injuries remains unclear. The current population-based study aimed to fill this gap by assessing the association between the use of ADHD medication - overall and specifically for stimulants and non-stimulants - and all-cause mortality, as well as unintentional injuries leading to ED and hospital admissions in children, adolescents, and young adults with ADHD. We hypothesize that after controlling for important potential confounders such as age, region of residence, area-level material and social deprivation, the number of consultations in the year prior to ADHD diagnosis or treatment with medication, as well as the presence of mental or substance use disorders and physical chronic conditions, the risk of all-cause mortality and unintentional injuries leading to ED or hospital admission will be lower during periods of ADHD medication use as opposed to episodes with no ADHD medication use.

## Methods

### Study population and data source

The source population consisted of children, adolescents, and young adults aged ≤ 24 years between 1 April 2000 and 31 March 2021 (*n* = 4,401,387), residing in the province of Quebec, Canada, where all residents are eligible for medical coverage under the provincial public health plan.

The Quebec Integrated Chronic Diseases Surveillance System (QICDSS) links, for the majority of residents (99%), data obtained from provincial health administrative databases on physician claims and diagnoses for medical services, pharmaceutical services dispensed in community pharmacies, hospital discharge diagnoses, vital statistics death registry, and health insurance eligibility (Supplementary information, page 1). In Quebec, drug insurance is mandatory, and residents must be covered under either a public or private drug insurance plan. Medications prescribed for individuals covered under a private drug plan are not recorded in the QICDSS database. The use of the QICDSS was approved by the Public Health Ethics Committee and the *Commission d’accès à l’information du Québec*, Quebec’s information and privacy commission.

### Study cohort

The study cohort (*n* = 217,192) included all residents aged ≤24 years who either had a physician claim or hospital diagnosis of ADHD (ICD-9 code: 314; ICD-10-CA codes: F900, F901, F908, F909) or had filled a prescription for an ADHD medication, which included amphetamine or methylphenidate-based stimulants and non-stimulants (atomoxetine and guanfacine), between 1 April 2000 and 31 March 2021. The cohort entry date was in relation to either the first physician diagnosis for ADHD or the first ADHD medication claim between 1 April 2000 and 31 March 2021. The end of the follow-up period was at emigration, death, age 25, or the end of the study period (31 March 2021).

### Study variables

The study outcomes included mortality from all causes, unintentional injuries leading to hospitalisations, and unintentional injuries leading to ED admissions. Information on the date of death was obtained from the vital statistics registry. ED medical claims with a diagnosis code related to injury and hospital accident codes were based on ICD-9 (800−949, 960−999) and their corresponding ICD-10-CA codes (V01−Y89) (Supplementary information, page 1).

The independent time-varying exposure of interest was defined as episodes of ADHD medication use and public drug insurance coverage. Episodes were defined according to the following mutually exclusive criteria: (1) no ADHD medication use while covered under the province’s public drug insurance plan; (2) ADHD medication use while covered under the public drug plan; and 3) not covered under the public drug plan. Episodes of ADHD medication use were further categorised as: either an amphetamine and/or methylphenidate-based stimulant only; a non-stimulant only; or a combination of an amphetamine and/or methylphenidate-based stimulant and, concomitantly, a non-stimulant. An episode was defined at the start of public drug coverage or loss of public drug coverage, the stop of an ADHD medication, a new ADHD medication filled (i.e., from non-use to ADHD medication use), a switch from an ADHD medication class to another (i.e., switch from a stimulant to a non-stimulant), or augmentation with another ADHD medication class (i.e., adding a non-stimulant to a stimulant regimen). A grace period of five days was allowed between the end of the supply date of an ADHD medication and the following ADHD medication dispensed. Also, a grace period of five days in the overlap between two different ADHD medications or classes was tolerated for switches; otherwise, the episode was considered as an add-on and classified as both stimulant and non-stimulant use.

Covariates at the time of study entry included demographic variables (sex, region of residence, material and social deprivation index) and the number of outpatient consultations in the year prior to study entry (either ADHD physician diagnosis or drug claim). The presence of a mental or substance use, endocrine, nervous system, or cardiovascular disorder, as well as congenital anomalies at any point during the study observation period was also controlled for. Age was considered time-dependent, and its time-varying effect was considered at the beginning of each episode. Covariate definitions are presented in Supplementary information (page 2).

### Statistical analyses

We reported descriptive statistics on the demographic and clinical characteristics of the study population (N, %). Unadjusted estimates for all-cause mortality and unintentional injuries leading to an ED admission or hospitalisation were presented as crude rates. The adjusted hazard ratios of all-cause mortality and injury associated with ADHD medication use were estimated by multivariable survival analyses using Prentice-Williams-Peterson (PWP) models, which allows for analysing recurrent events by ordering multiple events by stratification [31]. Two types of PWP models are available, one defined according to total time and one defined according to gap time since the previous event [31, 32]. Outcomes related to unintentional injuries leading to an ED admission or hospitalisation were studied with the PWP-gap time model, which evaluates the effect of a covariate for the kth event since the time of the (k-1) event. In the case of the gap time model, usually, each time interval starts at time zero and ends at the next event. The model was revised to set the time at 0 after each new exposure episode (i.e., ADHD medication use, drug insurance coverage) to better reflect the duration of action of ADHD medications since these medications need to be taken continuously to have a clinical effect. Therefore, each event of an injury or start of a new episode consisted of one stratum, with the risk of an injury being conditional on the number of prior exposure episodes and the prior number of injuries. All-cause mortality was studied using the PWP-total time model, which evaluates the effect of episodes of medication use since the time of study entry and where each new episode consisted of one stratum. Robust variance estimators were used to account for between-subject heterogeneity, and results were reported as hazard ratios (HRs) and 95% confidence intervals (CIs). Analyses were performed using SAS Enterprise Guide statistical software version 7.15. (SAS Institute).

### Sensitivity analyses

We conducted three sets of sensitivity analyses. First, to study the effects on all-cause mortality and injuries leading to an ED or hospital admission, we censored individuals at their loss of coverage under the public drug plan (i.e., switch to a private drug plan) (Supplementary Table 2, page 5). Second, we fitted the PWP-gap-time model with the classical definition that resets the time to 0 only after each event and subsequent recurrent event (unintentional injuries leading to ED or hospitalisation) with exposure episodes considered as time-dependent (Supplementary Table 3, page 5). Third, individuals entered the study cohort only at the first ADHD diagnosis (Supplementary Table 4, page 6) and had to be covered under the public drug insurance plan in the 365 days prior and 183 days following study entry to assess the presence of an ADHD claim. Further, individuals having such events before study cohort entry were excluded from analyses to study the effects on incident injuries leading to an ED or hospital admission.

## Results

Characteristics of the 217 192 individuals in the study cohort are presented in Supplementary Table 1 (Supplementary information, page 3). Most individuals were males (64.1%), were aged below 11 years at first ADHD physician diagnosis or medication claim (64.2%) and had a comorbid mental or substance use disorder at study entry (78.5%).

The percent time of follow-up covered under the public drug plan with any ADHD medication was 22.0%, and specifically, 19.7% with any stimulant only, 1.2% with any non-stimulant only, and 1.1% with both stimulant and non-stimulant. Percent time of follow-up covered under the public drug plan without an ADHD medication was 51.6%, and percent time not covered under the public drug plan was 26.4%. The crude outcome rates are presented in Table 1.

**Table 1:** Crude rate of mortality, injuries leading to emergency department admissions and hospitalisations by ADHD medication episodes.

	Mortality			Injuries leading to ED admission			Injuries leading to hospitalisation		
	Number of events	Person-years	Crude rate per 1000 PY (95% CI)	Number of events	Person-years	Crude rate per 1000 PY (95% CI)	Number of events	Person-years	Crude rate per 1000 PY (95% CI)
Episodes with no ADHD medication use	474	979,214	0.48 (0.44–0.53)	96,160	977,823	98.3 (97.7–99.0)	8477	979,048	8.7 (8.5–8.8)
Episodes with ADHD medication use – Overall	109	416,848	0.26 (0.21–0.32)	37,915	416,778	91.0 (90.1–91.9)	3096	416,778	7.4 (7.2–7.7)
Episodes with ADHD stimulants only	96	373,514	0.26 (0.21–0.31)	33,854	373,453	90.7 (89.7–91.6)	2761	373,454	7.4 (7.1–7.7)
Episodes with ADHD non-stimulants only	10	22,753	0.44 (0.21– 0.81)	2254	22,744	99.1 (95.1–103.3)	174	22,744	7.7 (6.6–8.9)
Episodes with ADHD stimulants and non-stimulants	NR	NR	NR	1807	20,580	87.8 (83.8–92.0)	161	20,580	7.8 (6.7–9.1)
Not covered under public drug plan	247	500,632	0.49 (0.43–0.56)	51,735	500,632	103.3 (102.5–104.2)	4113	500,632	8.2 (8.0–8.5)

*PY* person-years, *NR* not reported.

NR: cells with less than 5 events cannot be published to respect INSPQ dissemination rules.

The average crude rates of all-cause mortality per 1000-person years during episodes with no ADHD medication use, ADHD medication use, and not covered under the public drug plan, were on average 0.48 (95% CI, 0.44–0.53), 0.26 (95% CI, 0.21–0.32), and 0.49 (95% CI, 0.43–0.56), respectively. The average crude rate per 1000-person years of injuries leading to ED was 98.3 (95% CI, 97.7–99.0) during episodes with no ADHD medication use, 91.0 (95% CI, 90.1–91.9) with ADHD medication use, and 103.3 (95% CI, 102.5–104.2) in those not covered under the public drug plan. The average crude rates per 1000-person years of injuries leading to hospitalisation during episodes with no ADHD medication use, ADHD medication use, and not covered under the public drug plan were 8.7 (95% CI, 8.5–8.8), 7.4 (95% CI, 7.2–7.7), and 8.2 (95% CI, 8.0–8.5), respectively.

The associations between ADHD medication use and all-cause mortality (Table 2) showed a decreased risk during episodes of ADHD medication use (aHR 0.61, 95% CI 0.48–0.76) compared to no ADHD medication use while covered under the public drug plan. The estimates were similar during episodes of stimulant use only (aHR 0.61, 95% CI 0.48–0.77), while no significant decrease was observed during episodes of non-stimulants, stimulants combined with non-stimulants, and non-coverage under the public drug plan compared to episodes with no ADHD medication use.

**Table 2:** Association between ADHD medication episodes and mortality.

	HR (95% CI)	aHR^a^ (95% CI)
Episodes with no ADHD medication use	1.00	1.00
Episodes with ADHD medication use – Overall	0.63 (0.50–0.78)	0.61 (0.48–0.76)
Episodes with ADHD stimulants only	0.62 (0.49–0.78)	0.61 (0.48–0.77)
Episodes with ADHD non-stimulants only	1.02 (0.55–1.89)	0.73 (0.39–1.37)
Episodes with ADHD stimulants and non-stimulants	0.32 (0.10–1.00)	0.33 (0.11–1.05)
Not covered under public drug plan	0.85 (0.72–1.01)	1.04 (0.88–1.24)

^a^*aHR* adjusted for age, sex, past-year number of outpatient physician consultations at study cohort entry; the presence of mental and substance use disorders, and the presence of endocrine, nervous system, and cardiovascular disorders, congenital anomalies during observation period; region of residence; social and material deprivation index.

Table 3 shows the association between ADHD medication use and unintentional injuries leading–an ED admission. There was a decreased risk of unintentional injuries during episodes of ADHD medication use (aHR 0.75, 95% CI 0.74–0.77) as compared to no ADHD medication use while covered under the public drug plan. The results were similar during episodes of stimulant (aHR 0.76, 95% CI 0.75–0.77), non-stimulant (aHR 0.77, 95% CI 0.73–0.81) and combined stimulant and non-stimulant use (aHR 0.66, 95% CI 0.62–0.70). The risk of injury leading to an ED admission during episodes not covered under the public drug plan was higher than during episodes with no ADHD medication use while covered by the drug plan.

**Table 3:** Association between ADHD medication episodes and unintentional injuries leading to emergency department admission.

	HR (95% CI)	aHR^a^ (95% CI)
Episodes with no ADHD medication use	1.00	1.00
Episodes with ADHD medication use – Overall	0.75 (0.74–0.76)	0.75 (0.74–0.77)
Episodes with ADHD stimulants only	0.75 (0.74–0.76)	0.76 (0.75–0.77)
Episodes with ADHD non-stimulants only	0.81 (0.77–0.86)	0.77 (0.73–0.81)
Episodes with ADHD stimulants and non-stimulants	0.66 (0.62–0.70)	0.66 (0.62–0.70)
Not covered under public drug plan	1.05 (1.03–1.06)	1.09 (1.08–1.11)

^a^*aHR* adjusted for sex, past-year number of outpatient physician consultations at study cohort entry; the presence of mental and substance use disorders, and the presence of endocrine, nervous system and cardiovascular disorders, congenital anomalies during observation period; region of residence; social and material deprivation index at study entry; and time varying factors such as age and prior number of unintentional injuries leading to ED admission.

The association between ADHD medication use and unintentional injuries leading to hospitalisation (Table 4) showed a decreased risk of unintentional injuries during episodes of ADHD medication use (aHR 0.71, 95% CI 0.68–0.75) compared to no ADHD medication use while covered under the public drug plan. The results were similar during episodes of stimulant (aHR 0.72, 95% CI 0.68–0.76), non-stimulant (aHR 0.66, 95% CI 0.57–0.78) and combined stimulant and non-stimulant use (aHR 0.68, 95% CI 0.57–0.82). There was no statistically significant association between injury-related hospitalisations and episodes not covered under the public drug plan compared to episodes with no ADHD medication use.

**Table 4:** Association between ADHD medication episodes and unintentional injuries leading to hospitalisation.

	HR (95% CI)	aHR^a^ (95% CI)
Episodes with no ADHD medication use	1.00	1.00
Episodes with ADHD medication use – Overall	0.72 (0.69–0.76)	0.71 (0.68–0.75)
Episodes with ADHD stimulants only	0.72 (0.69–0.76)	0.72 (0.68–0.76)
Episodes with ADHD non-stimulants only	0.75 (0.64–0.88)	0.66 (0.57–0.78)
Episodes with ADHD stimulants and non-stimulants	0.72 (0.60–0.86)	0.68 (0.57–0.82)
Not covered under public drug plan	0.94 (0.90–0.98)	0.99 (0.95–1.03)

^a^*aHR* adjusted for sex, past-year number of outpatient physician consultations at study cohort entry; the presence of mental and substance use disorders, and the presence of endocrine, nervous system and cardiovascular disorders, congenital anomalies during observation period; region of residence; social and material deprivation index at study entry; and time varying factors such as age and prior number of unintentional injuries leading to a hospitalisation.

The sensitivity analyses confirmed the robustness of the findings. The association between ADHD medication use and all-cause mortality and injuries leading to ED and hospital admissions where individuals were censored when they were no longer covered under the public drug plan showed similar results (Supplementary Table 2). Also, results did not change in the sensitivity analyses regarding PWP-gap time models for ED and hospital admissions (Supplementary Table 3). Similarly, findings were confirmed when restricting study cohort entry to an ADHD diagnosis only (Supplementary Table 4).

## Discussion

To our knowledge, this is the first study to investigate the impact of ADHD medications (stimulants and non-stimulants) on the risk of all-cause mortality and unintentional injuries while considering important potential confounders and the number of previous injuries leading to ED or hospital admissions. We found that stimulants were significantly associated with a reduced risk of all-cause mortality, which was not observed with the use of non-stimulants only or the combination of stimulants and non-stimulants. Additionally, using stimulants, non-stimulants or a combination of both was associated with a reduced risk of injuries leading to ED admissions or hospitalisations.

The current findings on mortality add to the recent literature showing that ADHD medications do not increase the risk of sudden deaths due to cardiovascular events [25, 33]. Therefore, our study contributes to the evidence that stimulants do not increase overall mortality risk but are associated with a decreased risk. The observed estimate of reduced risk of mortality associated with stimulants was similar to estimates previously reported on the risk of all-cause mortality associated with the use of methylphenidate for the treatment of ADHD [24]. Our findings also align with the results of a recent national registry study among individuals with a mental disorder related to methamphetamine or amphetamine use disorder. The study found that using methylphenidate was associated with a similar reduced risk of all-cause mortality, while atomoxetine was not [34].

Possible reasons for the reduced risk of mortality associated with ADHD medication use in children with ADHD include a decreased risk of impulsive behaviours leading to unintentional physical injuries [27] and motor vehicle accidents [26], as well as brain injuries leading to hospitalisations [35], which may account for the increased all-cause mortality.

Notably, none of the previous studies reported on mortality associated with the use of non-stimulants specifically, as most participants were prescribed stimulants. At least two explanations exist for the lack of association between reduced mortality risk and episodes of non-stimulant use alone or in combination with stimulants. First, at the group level, non-stimulants are less effective than stimulants in treating ADHD [36] and take longer to be effective [37], which may confer less protection against injuries leading to mortality. Second, as per current guidelines, non-stimulants are usually prescribed to individuals who have not responded to or tolerated stimulants or to individuals at risk for substance dependence and those with psychiatric comorbidity [30]. Therefore, this population may be characterised by a higher mortality risk and may be less responsive to the beneficial effects of medications.

The current study findings on the risk of injuries leading to ED or hospital admissions add to the few available within-individual study designs, which show that ADHD stimulant treatment is associated with a reduced risk of injuries leading to ambulatory and inpatient visits in children, adolescents, and young adults with ADHD [27, 38]. To our knowledge, none of the available studies has assessed the association between non-stimulant use alone or co-prescribed with stimulants and the risk of unintentional injuries. A possible explanation for the protective effect of stimulants and non-stimulants includes, in addition to their effects in terms of reducing impulsivity, their effectiveness in improving symptoms of aggression and conduct, as well as oppositional behaviours [39].

The risk of all-cause mortality and unintentional injuries leading to hospitalisation was not significantly different during episodes where individuals were not covered under the public drug plan compared to those covered by the public drug plan without using ADHD medications. The sensitivity analysis, however, on incident injuries leading to hospitalisation in the cohort defined on an ADHD diagnosis at study entry showed a lower risk of hospitalisation. However, the risk leading to ED admissions during episodes not covered under the public drug plan was significantly higher than during episodes with no ADHD medication use while covered by the drug plan. According to available data from provincial private insurance drug plans, the most widely prescribed medication was long-acting methylphenidate, including its generic formulations, followed by long-acting amphetamines, non-stimulants (atomoxetine and guanfacine) and, short and intermediate acting stimulants [40]. Furthermore, based on available data, the number of long-acting stimulant and non-stimulant prescriptions delivered under private drug insurance plans seem higher than under the public drug insurance plan [40]. However, these findings cannot be directly extrapolated to the current population with private insurance, as information on medication use was not captured during these periods. Alternatively, the findings may reflect an overall average effect between medication and non-medication use episodes. The sensitivity analyses showed similar results.

The current study should be considered in light of some limitations. First, the cohort was built by including individuals with either an ADHD medical diagnosis or medication claim. This definition of cohort entry overcomes limitations of provincial health administrative databases where physicians are not obliged to submit a diagnostic code for payment. However, by including individuals based on medication claims, we may have selected more severe ADHD cases necessitating treatments. Nonetheless, importantly, the sensitivity analysis we performed selecting individuals based only on ADHD diagnosis gave similar results, suggesting that any possible selection bias was minimal. Additionally, the definition of exposure considered not only medications but also the coverage under the public drug plan, creating an exposure class for which medication use was not ascertainable. Therefore, the study design allowed for outcome risk ascertainment during periods when individuals were not covered under the public drug plan, thus reducing the potential selection bias that would have occurred by excluding individuals not covered under the public drug plan during the entire study period. Besides, the sensitivity analysis conducted by censoring individuals at the loss of their coverage under the public drug plan did not yield different results. Although the current analyses controlled for several socio-demographic and clinical factors, residual confounding cannot be excluded. The current study focused on children and young adults aged 24 years and under. Based on surveys and stakeholder consultations, Quebec’s health technology agency reported that very few psychosocial services are offered in the province of Quebec to residents aged 25 years and less with ADHD [41], whereby the majority of treatment includes pharmacotherapy. Focusing on this age range also allowed us to minimise any misclassification bias related to psychosocial and psychotherapy treatments for ADHD, that are not accurately captured in Quebec’s medical claims database. Similar population-based studies in different health system contexts are needed to improve the generalizability of results to other population groups.

## Conclusion

This is the first study showing a reduced risk of unintentional injuries leading to emergency department visits and hospitalisations associated with ADHD medication and a reduced risk of all-cause mortality, particularly with the use of stimulants rather not non-stimulants. Future studies should focus specifically on the long-term effects of ADHD medication use initiated in childhood, adolescence, and young adulthood on traumas and premature death into adulthood. This may better elucidate the impact of ADHD medication use from a life-course perspective.

### Supplementary information

Supplement

## Data Availability

The authors are not legally authorised to share or publicly publish health administrative data due to privacy or ethical restrictions related to the use of administrative provincial health data. The province of Québec’s ‘*Commission d’Accès à l’Information’* did not give approval to share these data. Requests for access to the data should be addressed to the *Institut National de Santé Publique du Québec*.
