# Supplementary material for: ADHD medications use and risk of mortality and unintentional injuries: a population-based cohort study
Source: Transl Psychiatry. 2024 Feb 28;14:128. doi: 10.1038/s41398-024-02825-y (PMC10901868; doi:10.1038/s41398-024-02825-y)
Supplement: Supplementary file 1 — Supplement [file 41398_2024_2825_MOESM1_ESM.docx]

## Supplementary information

## This file presents supplementary information on the methods used, study sample characteristics and sensitivity analyses carried out.

**Quebec Integrated Chronic Diseases Surveillance System (QICDSS)**

The QICDSS links outpatient, inpatient and pharmaceutical registry health administrative databases [1-3]. The *Régie de l’Assurance Maladie du Québec* (RAMQ) (Quebec’s National Health Insurance Plan) holds data on physician billing. It includes the date of service, diagnosis, specialty and setting where the act took place. The majority of physicians in Quebec work and bill the public sector [1, 2]. The *Maintenance et exploitation des données pour l’étude de la clientèle hospitalière* (MED-ECHO) (Maintenance and exploitation of data for the study of hospitalised clientele) database on hospitalisations includes diagnostic codes based on the International Classification of Diseases 9^th^ revision (ICD‐9) up to March 31, 2006, and the 10^th^ revision (ICD-10-CA) thereafter. The RAMQ pharmaceutical services registry holds information on all medications dispensed in ambulatory settings to residents covered under the public drug plan.

**Drug insurance plan in the province of Quebec^*^**

By law, residents must hold private or public drug insurance to cover medication costs. Medications prescribed during periods covered under a private drug plan are not recorded in the QICDSS database; as such, usually, these individuals are excluded by studies using this administrative database. The definition of exposure in the current study addressed this real-world context by considering an individual’s risk of outcomes during episodes of not being covered under the public drug plan. Episodes where individuals are covered under the private drug plan usually represent periods where children, adolescents and young adults are covered under their parents’ private insurance plans or have their own through academic institutions or employers. An individual without private insurance becomes covered by the public drug plan, and when they acquire private insurance, they are no longer covered under the public plan.

^*^ <https://www.ramq.gouv.qc.ca/en/citizens/prescription-drug-insurance/obligation>

**Study outcomes: unintentional injury leading to ED or hospital admission**

An unintentional injury leading to an ED admission [ICD-9 codes: 800−949, 960−999] was identified from medical claims in the RAMQ fee-for-service physician registry file. Medical claims for procedures in the ED billed during consecutive days were considered as one ED admission. A minimum period of 30 days between medical claims was used to distinguish between two unrelated unintentional injuries leading to a new ED admission.

An unintentional injury leading to hospital admission [ICD-10-CA codes: V01−Y89] was identified from the MED-ECHO database. An ED admission leading to hospitalisation was not considered as a separate ED admission event. That is, for individuals admitted to hospital from the ED, the event considered would be the hospital stay and not the ED.

**Covariate definitions**

Region of residence was categorised according to four categories: 1) Montreal census metropolitan area (CMA), 2) CMA ≥ 100,000 inhabitants (large urban cities), 3) CMA 10,000 – 99,999 inhabitants (smaller urban cities), and 4) rural areas (Statistics Canada). Individual level socioeconomic indicators were not captured in the CIQDSS. However, estimates of the socioeconomic level of the area of residence were based on geographic indices of social and material deprivation according to 2016 census data [4]. Data on material deprivation were based on area-level income, education and employment status, whereas information on social deprivation was based on single-resident homes, single persons and single-parent families. Material and social deprivation quintiles were ordered from the least deprived (1^st^ quintile) to the most deprived (5^th^ quintile). During the observation period, individuals with a mental (excluding ADHD diagnosis) or substance use disorder were identified with the corresponding ICD-9 (290−313; 315−319) and ICD-10 (F00−F89; F91−F99) codes, as was the presence of endocrine (ICD-9: 240−279; ICD-10: E00−E99) and nervous (ICD-9: 320−349; ICD-10: G00−G49, G80−G99) system disorders, cardiovascular disorders (ICD-9: 390−459; ICD-10: I00−I99) and congenital anomalies (ICD-9: 740−759; ICD-10: Q00−Q99).

**Study sample characteristics:**

**Table 1. Demographic and Clinical Characteristics of individuals with ADHD or ADHD medication claim in Quebec between 2000 and 2021 (n=217 192)**

| Study variables | N (%) |
| --- | --- |
| ADHD physician diagnosis and medication claim |  |
| ADHD physician claim only  ADHD medication claim only  ADHD physician and medication claims | 29 886 (13.8%)  52 487 (24.2%)  134 819 (62.0%) |
| Age |  |
| 1−11 years | 139 417 (64.2%) |
| 12−17 years | 39 649 (18.3%) |
| 18−24 years | 38 126 (17.6%) |
| Sex |  |
| Female | 77 859 (35.9%) |
| Male | 139 333 (64.1%) |
| Region of residence |  |
| Census metropolitan area of Montreal | 89 935 (41.4%) |
| Census metropolitan area with ≥ 100,000 inhabitants | 41 609 (19.2%) |
| Census metropolitan area with 10,000 – 99,999 inhabitants | 32 115 (14.8%) |
| Rural areas ≤9,999 | 53 103 (24.5%) |
| Missing | 430 (0.2%) |
| Area level of residence material deprivation |  |
| Quintile 1 (Least deprived) | 27 539 (12.7%) |
| Quintile 2 | 35 614 (16.4%) |
| Quintile 3 | 41 873 (19.3%) |
| Quintile 4 | 48 425 (22.3%) |
| Quintile 5 (Most deprived) | 56 442 (26.0%) |
| Missing | 7 299 (3.4%) |
| Area level of residence social deprivation |  |
| Quintile 1 (Least deprived) | 36 859 (17.0%) |
| Quintile 2 | 41 178 (19.0%) |
| Quintile 3 | 42 270 (19.5%) |
| Quintile 4 | 42 453 (19.6%) |
| Quintile 5 (Most deprived) | 47 133 (21.7%) |
| Missing | 7 299 (3.4%) |
| Mental and substance use disorder (not ADHD)  No | 46 598 (21.5%) |
| Yes | 170 594 (78.5%) |
| Depressive disorder | 35 123 (16.2%) |
| Anxiety disorder | 10 802 (5.0%) |
| Substance use disorder | 23 401 (10.8%) |
| Autism | 12 893 (5.9%) |
| Conduct or behavioural disorder in childhood and adolescence | 102 577 (47.2%) |
| Adjustment disorders | 60 098 (27.7%) |
| MD including Bipolar disorder, Psychosis, Schizophrenia | 17 127 (7.9%) |
| Mental retardation | 7 166 (3.3%) |
| Endocrine system disorder  No | 167 194 (77.0%) |
| Yes | 49 998 (23.0%) |
| Nervous system disorder  No | 181 358 (83.5%) |
| Yes | 35 834 (16.5%) |
| Cardiovascular disorders  No | 178 821 (82.3%) |
| Yes | 38 371 (17.7%) |
| Congenital anomalies  No | 174 874 (80.5%) |
| Yes | 42 318 (19.5%) |
| Outpatient claims in the 12 months prior of study entry |  |
| 0  1  2  3  4  5  6  7+ | 34 598 (15.9%)  41 457 (19.1%)  35 020 (16.1%)  26 857 (12.4%)  19 912 (9.2%)  14 496 (6.7%)  10 473 (4.8%)  34 379 (15.8%) |

**Sensitivity analyses:**

1) Sensitivity analyses with censoring at loss of public drug insurance plan coverage (i.e. switch to a private drug plan).

Supplementary Table 2. **Association Between ADHD Medication Episodes and All-Cause Mortality, Unintentional Injuries leading to ED admission or Hospitalisation^a^**

|  | **All-cause mortality** | **ED admission** | **Hospitalisation** |
| --- | --- | --- | --- |
|  | **aHR (95% CI)** | **aHR* (95% CI)** | **aHR** (95% CI)** |
| Episodes with no ADHD medication use | 1.00 | 1.00 | 1.00 |
| Episodes with ADHD medication use – Overall | 0.59 (0.46 to 0.77) | 0.75 (0.73 to 0.76) | 0.72 (0.68 to 0.77) |
| Episodes with ADHD stimulants only | 0.61 (0.47 to 0.80) | 0.75 (0.74 to 0.76) | 0.72 (0.68 to 0.77) |
| Episodes with ADHD stimulants only | 0.60 (0.28 to 1.32) | 0.76 (0.71 to 0.81) | 0.68 (0.57 to 0.82) |
| Episodes with ADHD stimulants and non-stimulants | 0.26 (0.06 to 1.11) | 0.66 (0.61 to 0.71) | 0.71 (0.58 to 0.88) |
| **^a^** Cohort is censored at the earliest date of either outcome, no longer covered under the public drug plan, emigration, age 25 or end of study.  **aHR:** Adjusted for sex, past-year number of outpatient physician consultations at study cohort entry; the presence of mental and substance use disorders, and the presence of endocrine, nervous system and cardiovascular disorders, congenital anomalies during observation period; region of residence, social and material deprivation index at study entry, and time varying factors such as age and *prior number of unintentional injuries leading to ED admission; ** leading to a hospitalisation. | | | |

2) PWP-gap time model where each time interval starts at time zero and ends until the next event, after which the time is reset at 0, and this at each recurrent event [5-7].

Supplementary Table 3. **Association Between ADHD Medication Episodes and Unintentional Injuries leading to ED admission or Hospitalisation**

|  | **ED admission** | **Hospitalisation** |
| --- | --- | --- |
|  | **aHR* (95% CI)** | **aHR** (95% CI)** |
| Episodes with no ADHD medication use | 1.00 | 1.00 |
| Episodes with ADHD medication use – Overall | 0.80 (0.79 to 0.82) | 0.73 (0.70 to 0.77) |
| Episodes with ADHD stimulants only | 0.80 (0.79 to 0.81) | 0.73 (0.69 to 0.77) |
| Episodes with ADHD non-stimulants only | 0.85 (0.81 to 0.89) | 0.70 (0.60 to 0.82) |
| Episodes with ADHD stimulants and non-stimulants | 0.82 (0.78 to 0.87) | 0.79 (0.66 to 0.94) |
| Not covered under public drug plan | 1.12 (1.11 to 1.13) | 1.01 (0.97 to 1.05) |
| **aHR:** Adjusted for sex, past-year number of outpatient physician consultations at study cohort entry; the presence of mental and substance use disorders, and the presence of endocrine, nervous system and cardiovascular disorders, congenital anomalies during observation period; region of residence, social and material deprivation index at study entry, and time varying factors such as age and *prior number of unintentional injuries leading to ED admission; ** leading to a hospitalisation. | | |

3) Sensitivity analyses were restricted to individuals who entered the study at a first ADHD diagnosis between April 1^st^, 2000 and March 31^st^, 2021. Individuals had to be covered under the public drug insurance plan in the 365 days prior and 183 days following study entry. Individuals having received an ADHD medication prior to diagnosis were excluded. Individuals with injuries leading to ED admission and hospital admission prior to study cohort entry were excluded from the analyses on the effects on injuries leading to ED admission and hospital admission, respectively. End of follow-up was at emigration, death, age 25, or end of study, March 31^st^, 2021.

Supplementary Table 4. **Association Between ADHD Medication Episodes and All-cause Mortality, Unintentional Injuries leading to ED admission or Hospitalisation**

|  | **Mortality** | **ED admission** | **Hospitalisation** |
| --- | --- | --- | --- |
|  | **aHR (95% CI)** | **aHR* (95% CI)** | **aHR** (95% CI)** |
| Episodes with no ADHD medication use | 1.00 | 1.00 | 1.00 |
| Episodes with ADHD medication use – Overall | 0.68 (0.46 to 0.99) | 0.73 (0.70 to 0.75) | 0.68 (0.63 to 0.74) |
| Episodes with ADHD stimulants only | 0.75 (0.50 to 1.10) | 0.73 (0.70 to 0.75) | 0.68 (0.63 to 0.74) |
| Episodes with ADHD non-stimulants only | 0.72 (0.23 to 2.29) | 0.79 (0.71 to 0.88) | 0.63 (0.49 to 0.81) |
| Episodes with ADHD stimulants and non-stimulants | - | 0.68 (0.61 to 0.76) | 0.73 (0.58 to 0.93) |
| Not covered under public drug plan | 1.18 (0.86 to 1.62) | 1.05 (1.02 to 1.08) | 0.91 (0.84 to 0.98) |
| **aHR:** Adjusted for sex, past-year number of outpatient physician consultations at study cohort entry; the presence of mental and substance use disorders, and the presence of endocrine, nervous system and cardiovascular disorders, congenital anomalies during observation period; region of residence, social and material deprivation index at study entry, and time varying factors such as age and *prior number of unintentional injuries leading to ED admission; ** prior number of hospitalisations. | | | |

## References

1. Contandriopoulos D, Law MR. Policy changes and physicians opting out from Medicare in Quebec: an interrupted time-series analysis. *Cmaj* 2021; 193: E237-e241.

2. An overview of physician payments and cost per service. <https://www.cihi.ca/en/health-workforce-in-canada-in-focus-including-nurses-and-physicians/an-overview-of-physician>, 2022, Accessed Date Accessed 2022 Accessed.

3. Blais C, Jean S, Sirois C, Rochette L, Plante C, Larocque I et al. Quebec Integrated Chronic Disease Surveillance System (QICDSS), an innovative approach. *Chronic Dis Inj Can* 2014; 34: 226-235.

4. Material and social deprivation index. <https://www.inspq.qc.ca/en/deprivation/material-and-social-deprivation-index>, 2019, Accessed Date Accessed 2019 Accessed.

5. Prentice RL, Williams BJ, Peterson AV. On the regression analysis of multivariate failure time data. *Biometrika* 1981; 68: 373-379.

6. Amorim LD, Cai J. Modelling recurrent events: a tutorial for analysis in epidemiology. *Int J Epidemiol* 2015; 44: 324-333.

7. Lu J, Shen D. Application of survival analysis in multiple events using SAS. PharmaSUG2018: Seattle, Washington, 2018.
